# Editorial: Environmental factors implicated in obesity

**DOI:** 10.3389/fnut.2023.1171507

**Published:** 2023-05-04

**Authors:** Ludovica Verde, Evelyn Frias-Toral, Diana Cardenas

**Affiliations:** ^1^Department of Public Health, University of Naples Federico II, Naples, Italy; ^2^School of Medicine, Universidad Católica Santiago de Guayaquil, Guayaquil, Guayas, Ecuador; ^3^Faculty of Medicine, Research Institute on Nutrition, Genetics and Metabolism, El Bosque University, Bogotá, Colombia

**Keywords:** obesity, environment, gut microbiota, food insecurity, stress

The prevalence of obesity has considerably grown in the past few decades globally. Several environmental and behavioral risk factors could explain, at least in part, this observed increase. Obesity predisposes to several comorbidities and a higher risk of mortality. In particular, Cai et al. found that adiposity was associated with a higher risk for all-cause mortality, assessed with the novel index termed the weight-adjusted waist index (WWI). Modern lifestyles are characterized by a variety of potential factors contributing to the development of obesity, such as poor diet quality due to increased exposure to junk food and the scarcity of healthy alternatives, especially in some places (so-called food deserts), but also higher levels of stress resulting from modern life (1). People living in food deserts may rely on unhealthy, processed, and high-calorie foods readily available in convenience stores and fast-food restaurants. This limited access to healthy food options and reliance on unhealthy foods can contribute to the development of obesity and diet-related chronic diseases. The absence of availability of nutritious dietary alternatives can lead to a diet high in calories, saturated fats, and added sugars, which can result in weight gain and the development of obesity. Moreover, food deserts are often found in low-income communities, where residents may face additional barriers, such as transportation difficulties and financial constraints, that limit their ability to access healthy food (2). These factors can lead to a higher reliance on unhealthy meals and play a role in increasing obesity rates.

Furthermore, the transition from healthy dietary patterns, characterized by a preference for plant-based foods, to a Western-style diet, marked by high consumption of nutrient-poor and energy-dense ultra-processed foods such as sugar-sweetened drinks (SSBs), has been identified as a possible cause (3, 4). Ultra-processed foods are typically high in calories and low in nutrients and have been linked to an increased risk of type 2 diabetes, obesity, and other chronic conditions (5). Their consumption has been on the rise globally, and this trend has been associated with adopting Western-style dietary patterns. These diets frequently contain little vegetables, fruits, and whole grains and are dominated by high consumption of high-fat snacks, processed fast foods, and SSBs. The high availability and low cost of ultra-processed foods and aggressive marketing strategies have contributed to their widespread consumption. Therefore, reducing their intake is essential to promoting healthy dietary patterns. Interventions to reduce the consumption of ultra-processed foods can include policies that limit the marketing and availability of these products, education campaigns that promote the consumption of whole, minimally processed foods, and initiatives that improve access to healthy foods in low-income communities. By addressing the role of ultra-processed foods in unhealthy dietary patterns, we can help promote healthier eating habits and reduce the burden of chronic diseases.

The link between diet quality and obesity is quite complex and most likely multidimensional, involving other aspects of lifestyle (e.g., level of physical activity, feeding times, chronotype, smoking, and alcohol consumption habits, etc.) as well as certain genetic predispositions (6). Of note, the genetics and genomics of nutrition are tools that form the basis for understanding the genetic pathways that are influenced by diet and lead to an increased predisposition to obesity. In addition to hormonal imbalances, alterations in the gut microbiota (GM) and obesity are also studied to unravel the mechanisms underlying the relationship between genetics, environment, GM, and obesity (4). Finally, the ever-increasing trends of obesity-related malignancies make this disease spectrum a public health priority (7). Research on this Research Topic is multidisciplinary. Therefore, future interventions for obesity will increasingly have to consider the numerous environment-obesity interactions. In this Research Topic, there are 10 papers covering the aspects mentioned above.

Food insecurity (FI), the lack of regular access to enough food for a healthy diet, has been the subject of two studies. Although the relationship between FI, poor diet quality, and obesity is widely established, more analysis of the underlying mechanisms and risks is required. Carvajal-Aldaz et al. noted that there are yet no mechanisms explaining this phenomenon. They concluded that although much evidence suggests a link between FI and obesity, this association has only been consistently seen in women from high-income countries, particularly the US. Future research must be adequately planned to shed light on the possible processes underlying this association. The same subject was covered by Fonseca-Pérez et al.; however, they focused on sarcopenic obesity. They reported that diet and aging-related impairments mediate the link between FI and sarcopenic obesity. Additionally, nutrition quality, a significant modifiable risk factor for the development of sarcopenia and obesity, can be influenced by FI. Noteworthy is the inverse relationship between diet quality and FI.

In the context of poor diet quality, SSBs, which contribute to excessive daily energy and sugar intake, are widespread worldwide. Mainly, SSB intake has been related to a higher risk of several health issues, including obesity, diabetes, and cardiovascular disease. Interesting research was conducted by AlFaris et al. on the consumption rates of weekly and daily SSBs in a multi-ethnic middle-aged men group and the relationships between obesity and sociodemographic factors. They discovered that nationality and obesity predicted both weekly and daily consumption of SSBs.

During the COVID-19 pandemic, Abril-Ulloa et al. investigated stress, another element linked to diet quality, among Ecuadorian adults. They demonstrated that higher stress levels were linked to worse diet quality and that the relationship between stress and diet quality was inverse and non-linear.

Thus, new public health measures in places (food deserts) where food insecurity predominates or at critical times (e.g., pandemics) are needed to improve the population's lifestyle and eating habits, which are well-known driving factors for obesity. In this regard, Limone et al.'s notable research has made a major contribution to the current state of information in serious games as a strategy to solve the problem of unhealthy eating behavior. They demonstrated that several serious game projects are effective interventions to modify the eating behavior of children and adults to address the risks of obesity and overweight in these populations.

Also, modern technologies can come to the rescue. In the future, it is intended to achieve personalized customization of the nutritional requirements of different populations and individuals based on the genetic inheritance of variants, ethnicity, and gene expression. The review by Guevara-Ramírez et al. synthesizes dietary practices in Latin America and the relationships between genes and single nucleotide polymorphisms (SNPs) linked to obesity, including the risk allele frequencies. They concluded that several genes and their SNPs had been associated with obesity and obesity-related issues. The risk alleles have been correlated with the deterioration of the lipid profile, and high-fat dietary behaviors were found to induce gene expression profiles related to several metabolic alterations. In addition, in the last few years, increasing evidence linking obesity to GM has been reported. GM management has become a new method of obesity treatment. Sarmiento-Andrade et al. have summarized the biology and physiology of GM in obesity, its role in the pathophysiology of several obesity-related disorders, and the emerging therapeutic applications of prebiotics, probiotics, and fecal microbiota transplantation. Instead, Mayorga-Ramos et al. focused their research on the exciting role of butyrate (a short-chain fatty acid produced by GM) in obesity and diabetes. The authors, after an exhaustive review of the literature, point out that efforts are still needed to decipher well the determination of the best conditions and food sources for butyrate production by the GM *in situ*, the absorption of dietary and microbially produced butyrate under different physiological and pathological conditions, the regulatory mechanisms of butyrate at the cellular and systemic level, and the potential for use as a therapeutic alternative in specific obesity-related disorders. Overall, the connection between genetics, gut microbiota, and obesity related-environmental factors is complex and interrelated. While genetics play a role in determining an individual's susceptibility to obesity, environmental factors such as diet, physical activity, and exposure to endocrine disruptors can influence the gut microbiota and contribute to the development of obesity.

Finally, starting from the evidence that the Mediterranean diet (MD) is a healthy diet effective in tackling obesity and its consequences, Barrea et al. demonstrated that low adherence to the MD was also associated with the presence of nodular thyroid disease and in particular with those at high risk of malignancy in a cohort of subjects with overweight/obesity. These results underscore the importance of promoting healthy dietary habits and adherence to MD as a preventative measure against cancers and associated health risks.

In summary, the reviews and studies mentioned earlier represent a broad amount of new relevant data on the environmental and behavioral factors involved in the etiology, pathophysiology, and treatment of obesity. The articles included in this Research Topic demonstrate that many parts of the issue still need to be defined and understood, despite all the research and data that already exist on this crucial subject. After reading this Research Topic, some topics, such as environmental factors involved in the development and treatment of obesity, will appear more clear/evident to the reader and strengthen the conviction that environmental intervention is a fundamental part of obesity prevention and treatment.

## Author contributions

LV wrote the first draft of the manuscript. All authors contributed to manuscript revision, read, and approved the submitted version.
